# Response to HER2 Inhibition in a Patient With Brain Metastasis With EGFR TKI Acquired Resistance and an HER2 Amplification

**DOI:** 10.3389/fonc.2018.00176

**Published:** 2018-05-22

**Authors:** Arenda D. Meedendorp, Arja ter Elst, Nils A. ’t Hart, Harry J. M. Groen, Ed Schuuring, Anthonie J. van der Wekken

**Affiliations:** University of Groningen and University Medical Center Groningen, Groningen, Netherlands

**Keywords:** EGFR, HER2, non-small-cell lung carcinoma, TKI, brain metastasis, Molecular Tumor Board

## Abstract

A 62-year-old man was referred to our university hospital for treatment of advanced adenocarcinoma of the lung after disease progression on two lines of EGFR TKI and one line of chemotherapy. Fluorescent *in situ* hybridization analysis upon progression showed an *HER2* amplification. At our weekly Molecular Tumor Board (MTB), a decision was made to treat this patient with afatinib, which resulted in a partial response. However, progression was observed with a facial nerve paresis due to a metastasis in the skull. A biopsy of a location in the thorax revealed the presence of an EGFR-T790M mutation associated with acquired resistance, after which treatment with osimertinib was started. After 6 months, disease progression was observed, and a new biopsy was taken from the pelvic bone, which revealed the original amplification of *HER2* together with the EGFR-L858R mutation, the EGFR-T790M mutation was not detected. The MTB decided to treat the patient with trastuzumab/paclitaxel. A partial response was observed in different bone lesions, while the skull metastasis with ingrowth in the brain remained stable for 6 months. Because of progression of the bone metastases after 6 months, a biopsy of a lesion in the thorax wall was taken. In this lesion, the EGFR-T790M mutation could be detected again. The MTB advised to start treatment with a combination of osimertinib and afatinib. This resulted in an impressive clinical improvement and a partial response of the bone metastases on the most recent 18-fluorodeoxyglucose positron emission tomography and computer tomography-scan. In conclusion, adjusting treatment to the mutational make-up of the tumor is a great challenge. For optimal treatment response multiple biopsies and re-biopsy upon progression are imperative. As more genes are investigated, treatment decision becomes increasingly difficult, therefore, expert opinions from an MTB is essential.

## Introduction

Treatment of driver mutations cannot be based on large clinical trials or high levels of evidence at all times. However, a Molecular Tumor Board (MTB) can help in making treatment decisions based on databases, case reports, xenograft models, and cell lines. Here, we present such a case.

**Figure 1:**
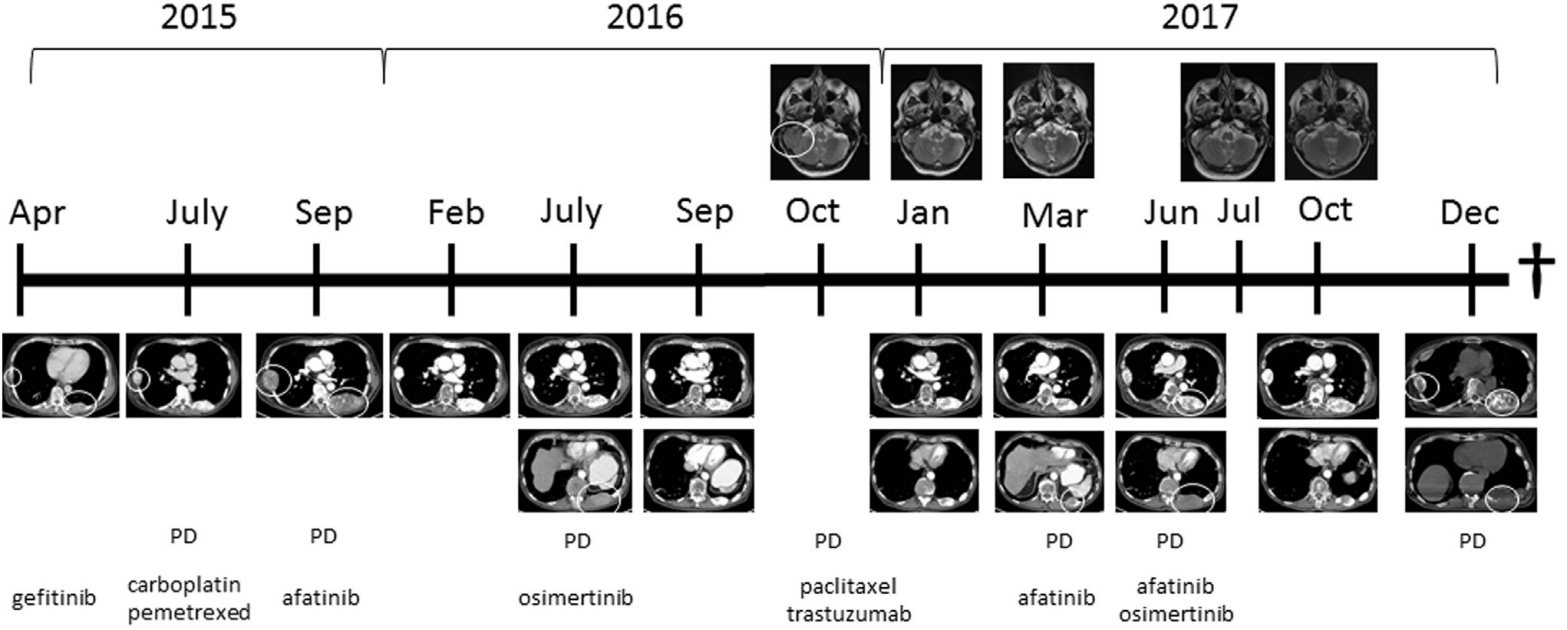
Computed tomography images showing the thoracic wall lesions of the adenocarcinoma of the lung and MRI images showing the metastasis in the skull with ingrowth in the brain at different time points in combination with treatment started at those time points. Abbreviation: PD, progressive disease. White circles indicate lesion, which showed progression.

A 62-year-old man was referred to our university hospital for treatment of advanced adenocarcinoma of the lung after disease progression on two lines of EGFR TKI and one line of chemotherapy in September 2015 (Figure 1).

Four months prior, in April 2015, he was diagnosed with an adenocarcinoma of the left lung with multiple bone metastases in sternum, ribs, and vertebrae. A biopsy from a metastasis in the left femur showed a mutation in the *EGFR* gene: c.2573T>G; p.(L858R). He was initially treated with gefitinib. After 2 months, the patient showed progression of bone metastases; the same EGFR mutation was found in a biopsy of a rib metastasis, without additional mutations in other mutational hotspots (e.g., *BRAF, KRAS, HER2, KIT, ALK, NRAS, PDGFRA, PIK3CA*, and *MET*). Therefore, at that time, carboplatin and pemetrexed were provided and because of pain, local irradiation of a sternal metastasis was applied. After two cycles of chemotherapy, the patient showed disease progression and was referred to our hospital. Because of lack of new treatment options, this patient was discussed in the Groningen MTB consisting of pulmonary oncologists, pathologists, clinical molecular biologists in pathology, general oncologists, and a structural biologist (www.moloncopath.nl). The MTB advised to determine the HER2-copy number status as a possible resistant mechanism for EGFR TKI. Fluorescent *in situ* hybridization (FISH) on a biopsy of a subcutaneous thoracic metastasis revealed *HER2* amplification and treatment with afatinib (dual EGFR and HER2 inhibitor) 30 mg QD was started in October 2015. Evaluation by 18-fluorodeoxyglucose positron emission tomography and computer tomography (18-FDG-PET-CT) showed after 6 weeks a significant partial response with disappearance of the FDG activity of the bone metastases and after 4 months in the left upper lobe a single FDG-positive lesion was left. This lesion was irradiated by means of stereotactic ablative radiotherapy (1× 20 Gy), and afatinib was continued. Treatment with afatinib was well tolerated with minor skin rash; patient showed clinical improvement: he had less pain and more energy. Nine months after start of afatinib, progressive disease was again noticed. Growth of the primary tumor in the left upper lobe, a new ipsilateral pulmonary lesion and multiple new bone metastases including the skull, with ingrowth into the brain, causing paralysis of the right facial nerve (Figure 1). Sequence analysis of a new right-sided rib lesion showed the known L858R *EGFR* mutation and an additional T790M mutation.

Because of the novel T790M, afatinib was discontinued and replaced by osimertinib 80 mg QD (1). Eight weeks after start of osimertinib a PET-CT showed a response of most lesions except for a growing lesion in the pelvic region and the skull with ingrowth in the brain. Clinically there was, however, temporary improvement of the patient’s ability to move his right eyelid and right corner of the mouth, which had been paralyzed due to ingrowth of a skull metastasis into the brain and right facial nerve. A biopsy was performed of a growing FDG-positive lesion in the left pelvic bone that showed adenocarcinoma with the known L858R *EGFR* mutation, but the previously found T790M mutation was not present in this location (no biopsy of the skull metastasis available). The MTB advised to perform immunohistochemistry on Her2Neu (positive in agreement with HER2 amplification) and to determine *MET* amplification (negative by FISH). Based on these findings, it was decided to discontinue osimertinib because of the loss of the T790M mutation and to start a combination of paclitaxel 90 mg/m^2^ on days 1, 8, and 15, and trastuzumab 4 mg/kg on days 1 and 15, in cycles of 4 weeks, because trastuzumab is an HER2 antibody. Radiotherapy 1× 8 Gy was given on the pelvic lesion because of localized pain. 18-FDG-PET-scan after four cycles, paclitaxel and trastuzumab showed again a partial tumor response. No major side effects were observed although symptoms of the paralysis of the right facial nerve did not improve further; it remained stable during the course of therapy. The patient underwent plastic surgery on his right eyelid, which improved the closure of his right eye.

Two months after the fourth and last cycle, the patient presented with a subcutaneous metastasis on his forehead. Afatinib 30 mg QD was started, because this treatment worked before, pending results of a new biopsy. The biopsy, however, yielded insufficient material for mutation analysis, and re-biopsy was scheduled. In the meantime, 18-FDG-PET-scan showed multiple FDG-positive bone lesions (partly new lesions), some close to the myelum, and the patient was admitted to the hospital for radiotherapy on cervical and thoracic vertebrae. Afatinib was discontinued. Biopsy of a new lesion in the thoracic wall showed an *EGFR*-L858R, T790M mutation, and *HER2* amplification. There were no other hotspot mutations in *EGFR, BRAF, KRAS, ERBB2 (HER2), ALK, PIK3CA*, or *MET* detected. The case was again reviewed by MTB. It was decided to treat the patient with afatinib 30 mg QD as well as osimertinib 80 mg QD at alternating days, to address the T790M mutation as well as the *HER2* amplification resistance mechanism. Since the start of this latest treatment regimen, the subcutaneous skull metastasis disappeared, and the patient experienced less pain, regained his energy, and was able to walk outdoors again. The most recent 18-FDG-PET-CT-scan, 4 months after the start of this latest treatment regimen, showed again a partial response of the bone metastases again. Two months later (December 2017) progression of disease was observed, and the performance status deteriorated. Patient insisted to take a new biopsy from a new thoracic wall metastasis. Mutations analysis showed the known *EGFR* L858R and T790M mutations together with a new mutation in *HER2*: L755S. However, his condition got worse in short time, and he died in January 2018. An overview of the clinical findings, the mutational status at different time points and the given treatment regimens is provided in Table 1.

**Table 1:** Overview of clinical and pathological findings and subsequent therapeutic decisions.

Clinical evaluation	Mutational status	CT/MRI evaluation	Therapy
Adenocarcinoma of the left lung with multiple bone metastases in sternum, ribs, and vertebrae	Femur: *EGFR*-L858R	April 2015	Gefitinib

Progression of bone metastases (time to progression: 2 months)	Rib: *EGFR*-L858R	July 2015	Carboplatinum and pemetrexed Irradiation on sternum

Progression of bone and subcutaneous metastases (time to progression: 2 months)	Thoracic subcutis: *HER2* amplification 3+	September 2015	Afatinib

Partial response with disappearance of the FDG activity of the bone metastases. Only 1 FDG-positive lesion in the left upper lobe	N.a.	March 2016	Stereotactic ablative radiotherapy of lesion left upper lobe Continuation of afatinib

Growth of primary tumor left upper lobe, ipsilateral pulmonary lesion, and multiple new bone metastases including the skull, with ingrowth into the brain (time to progression: 9 months)	Rib: *EGFR*-L858R and T790M	July 2016	Osimertinib

Mixed response: growing lesion left pelvic bone	Pelvic bone: *EGFR*-L858R, HER2 amplification 3+, no T790M	October2016	Paclitaxel and trastuzumab Irradiation on pelvic lesion

Subcutaneous metastasis on forehead and progression of bone metastases (time to progression: 6 months)	HER2 expression	April 2017	Afatinib Irradiation on cervical and thoracic vertebrae

N.a.	Thorax wall: *EGFR*-L858R and T790M mutation and *HER2* amplification 3+	June 2017	Afatinib and osimertinib

Partial response	N.a.	November 2017	Continuation of afatinib and osimertinib

Progressive disease	Thorax wall: *EGFR*-L858R, T790M, and HER2 L755S	December 2017	

Death		January 2018	

## Background

### EGFR

The incidence of *EGFR* mutations in advanced stage adenocarcinoma of the lung in Caucasian patients is 10–15 and 40–60% in Asian patients (2). In the north of the Netherlands, the incidence is 9% (3). L858R mutation in exon 21 of the EGFR kinase domain is the main hotspot mutation in the *EGFR* gene and accounts for 35–45% of *EGFR* mutations (4, 5). L858R mutation increases the kinase activity of EGFR, leading to hyperactivation of downstream signaling pathways improving cell survival and proliferation (6). An EGFR TKI is the preferred first-line treatment in patients with activating *EGFR* mutation in non-small-cell lung carcinoma (NSCLC) (4). Gefitinib and erlotinib are first-generation TKI and registered as first-line treatment in patients with metastatic NSCLC with a tumor harboring an activating *EGFR* mutation within the European Union and according to the Dutch guideline for treatment of NSCLC (7). These small molecules bind competitively and reversibly to the adenosine triphosphate (ATP) binding site of the tyrosine kinase domain of EGFR. This prevents the autophosphorylation of the TK, blocks the activation of the EGFR signal transduction, inhibits tumor cell proliferation, and induces cell cycle arrest and apoptosis (8). The majority of patients will progress after 9–12 months of treatment due to various mechanisms of intrinsic or acquired resistance to first-generation EGFR TKIs (9).

### EGFR T790M

The most common mechanism of acquired TKI resistance is the acquisition of a single recurrent missense mutation within exon 20, the T790M mutation (10). This mutation leads to the substitution of threonine by methionine at position 790, which encodes part of the kinase domain of the receptor and results in increased affinity for ATP (11). The T790M mutation can be detected in about 60% of tissue biopsy samples taken after acquired resistance (12, 13). As residue threonine at position 790 (T790) is located at the entrance in the back of the ATP binding cleft, substitution of residue threonine at position 790 with a bulky methionine (resulting in T790M) may cause steric interference with binding of TKIs (14). Irreversible inhibitors overcome this resistance simply through covalent binding (15). Osimertinib is registered for the treatment of NSCLC with an *EGFR* T790M mutation. It is a selective third-generation TKI which targets the ATP binding site of EGFR *via* irreversible covalent bond formation. In contrast to many other TKI, osimertinib penetrates the blood–brain barrier (16, 17). Osimertinib improves overall survival and progression-free survival in T790M-positive NSCLC patients with and without brain metastases (18, 19). Acquired resistance to osimertinib may be caused by primary coexistence of tumor cell populations with and without T790M mutation due to EGFR C797S mutation. Tumor progression can be explained by growth of the T790M negative population, while the tumor cells expressing T790M mutation are effectively suppressed by osimertinib (20).

### HER2

Overexpression of *HER2* induces cell transformation and tumorigenic growth and is clinically associated with resistance to erlotinib (21). *HER2* amplification is detected in a subset of EGFR TKI resistant lung tumors. *HER2* amplification and T790M mutation are thought to be mutually exclusive (22). However, in our patient *HER*2 amplification as well as T790M mutation appeared in the same biopsy of a new lesion in the thorax wall. Afatinib is an ATP-competitive aniline-quinazoline derivate which covalently binds to EGFR, HER2, and HER4 and irreversibly inhibits HER-family phosphorylation and signal transduction (23). As second generation TKI it is highly potent, irreversible dual EGFR/HER2 tyrosine kinase inhibitor, including the oncogenic *EGFR*-L858R mutation (23, 24). Afatinib is registered for advanced NSCLC with *EGFR* mutations. Clinical benefit of afatinib seems less in patients with *de novo* T790M mutations (25). Although afatinib is equally potent against wild-type EGFR and EGFR harboring the T790M mutation, in patients the dose is lower due to toxicity constraints (26).

Trastuzumab, a humanized monoclonal antibody against HER2, has been reported to be effective in *HER2*-positive NSCLC *in vitro* and in case reports (4, 27, 28).

## Discussion

Here, we describe a patient with EGFR mutant advanced NSCLC with recurrent episodes of disease progression due to subsequent mutant clones. Yu et al. selected 155 patients with lung adenocarcinomas and acquired resistance to erlotinib or gefitinib. These patients underwent a re-biopsy. The most common finding was a T790M mutation. They also found transformation to small cell lung carcinoma, *MET* amplification and *HER2* amplification (10). Sequist et al. described a wide variety of gained and lost *EGFR* mutations in a patient population with acquired drug resistance. They recommended reassessing cancers by taking new biopsies of growing lesions in patients with progressive disease after an initial response to TKI treatment (29). Following this strategy, we observed HER2 amplification and T790M mutation at different time points under the selective pressure of different EGFR TKI treatment. Of note, the occurrence of both aberrations at the same time has not been described earlier.

After discussion in the MTB about the most suitable therapy, as mentioned in the background, treatment was adjusted accordingly. Case evaluation by a multidisciplinary MTB is important to benefit from individualized genetic data and maximize clinical impact (30–32). MTB interprets results of routine molecular NGS-testing with those of other techniques, for example, immunohistochemistry, FISH, DNA methylation testing, and multiplex ligation-dependent probe amplification. NGS testing is not only performed on biopsies but currently also from tumor DNA in peripheral blood. The spectrum of molecular markers is constantly growing. Patients who progress after an EGFR TKI should undergo a re-biopsy to perform molecular analysis specifically looking for acquired mechanisms of resistance, such as *EGFR* T790M mutation. This approach can influence the next therapeutic step or reveal alternative EGFR TKI resistance mechanisms such as transformation to small cell lung cancer or bypass tracks that could potentially be addressed in clinical trials (11). Our patient responded well to subsequent treatment based on aberrations found in NGS, IHC, and FISH after discussion in the MTB. We observed that a change in treatment gave a short-lasting clinical improvement of several months and tumor response of fast growing new metastases. Osimertinib alternating with afatinib for T790M in EGFR and HER2 amplification was very effective in decreasing tumor sites. However, we expected that our patient would have immense toxicity of skin rash and diarrhea, but toxicity was not more than CTC grade 1. Long-lasting tiredness grade 1 was the most prominent side effect.

## Concluding Remarks

This case report shows the importance of re-biopsy of growing lesions in lung cancer patients with metastatic progressive disease under targeted therapies. Mutation status can vary under selection pressure of these drugs, and knowledge of these changes makes it possible to adapt treatments. This patient also exemplifies the importance of having a multidisciplinary expert team (MTB) to give rational treatment advice in cancer patients with uncommon mutations or combinations of mutations causing complex resistance mechanisms.

## Ethics Statement

This case report was written and offered for publication with written informed consent from the patient. The patient gave written informed consent in accordance with the Declaration of Helsinki.

## Author Contributions

AM: organized the relevant information about the patient; wrote all paragraphs of the article, did the literature research, and applied changes brought in by the other authors. AE, HG, and ES: supervised and corrected the manuscript. NH: supervised and gave advice. AW: main supervisor; supervised and corrected the manuscript.

## Conflict of Interest Statement

The authors declare that the research was conducted in the absence of any commercial or financial relationships that could be construed as a potential conflict of interest. The handling Editor declared a past co-authorship with one of the authors AW.
